# Impact of acute glycemic variability on short-term outcomes in patients with ST-segment elevation myocardial infarction: a multicenter population-based study

**DOI:** 10.1186/s12933-024-02250-x

**Published:** 2024-05-07

**Authors:** Juan Wang, Lu-lu Wang, Yan-min Yang, Hui-qiong Tan, Jun Zhu

**Affiliations:** 1 Emergency Center, National Clinical Research Center of Cardiovascular Diseases, Fuwai Hospital, National Center for Cardiovascular Disease, Chinese Academy of Medical Science and Peking Union Medical College, 100037 Beijing, China; 2 Intensive Care Center, National Clinical Research Center of Cardiovascular Diseases, Fuwai Hospital, National Center for Cardiovascular Diseases, Chinese Academy of Medical Sciences, Peking Union Medical College, 100037 Beijing, China; 3 Present Address: No.167 Beilishi Road, Xicheng District, 100037 Beijing, China

**Keywords:** Glycemic variability, ST-segment elevation myocardial infarction, Diabetes mellitus, Outcomes, All-cause death, Major adverse cardiovascular events

## Abstract

**Background:**

Given the increasing attention to glycemic variability (GV) and its potential implications for cardiovascular outcomes. This study aimed to explore the impact of acute GV on short-term outcomes in Chinese patients with ST-segment elevation myocardial infarction (STEMI).

**Methods:**

This study enrolled 7510 consecutive patients diagnosed with acute STEMI from 274 centers in China. GV was assessed using the coefficient of variation of blood glucose levels. Patients were categorized into three groups according to GV tertiles (GV1, GV2, and GV3). The primary outcome was 30-day all-cause death, and the secondary outcome was major adverse cardiovascular events (MACEs). Cox regression analyses were conducted to determine the independent correlation between GV and the outcomes.

**Results:**

A total of 7136 patients with STEMI were included. During 30-days follow-up, there was a significant increase in the incidence of all-cause death and MACEs with higher GV tertiles. The 30-days mortality rates were 7.4% for GV1, 8.7% for GV2 and 9.4% for GV3 (*p* = 0.004), while the MACEs incidence rates was 11.3%, 13.8% and 15.8% for the GV1, GV2 and GV3 groups respectively (*p* < 0.001). High GV levels during hospitalization were significantly associated with an increased risk of 30-day all-cause mortality and MACEs. When analyzed as a continuous variable, GV was independently associated with a higher risk of all-cause mortality (hazard ratio [HR] 1.679, 95% confidence Interval [CI] 1.005–2.804) and MACEs (HR 2.064, 95% CI 1.386–3.074). Additionally, when analyzed as categorical variables, the GV3 group was found to predict an increased risk of MACEs, irrespective of the presence of diabetes mellitus (DM).

**Conclusion:**

Our study findings indicate that a high GV during hospitalization was significantly associated with an increased risk of 30-day all-cause mortality and MACE in Chinese patients with STEMI. Moreover, acute GV emerged as an independent predictor of increased MACEs risk, regardless of DM status.

**Supplementary Information:**

The online version contains supplementary material available at 10.1186/s12933-024-02250-x.

## Background

Over the past few decades, there has been growing interest in glycemic variability (GV) [1]. Unlike mean blood glucose indices, GV represents fluctuations in glucose levels over various time intervals, encompassing both the amplitude and frequency of these fluctuations [2]. Elevated levels of GV, characterized by high frequency and amplitude fluctuations in blood glucose levels, have been implicated as potentially more detrimental than sustained hyperglycemia [3], and high GV has been identified as a contributing factor to the persistent high rates of complications associated with reaching target blood glucose levels [4]. Emerging research indicates that GV is an independent risk factor for diabetes mellitus (DM) complications, particularly in predicting cardiovascular risks [5]. Further investigations have established a correlation between acute GV and the severity and prognosis of various diseases, extending beyond DM [6] to encompass critically ill patients [7] and cardiovascular conditions [8].

Glycemic dysregulation is prevalent among patients diagnosed with ST-segment elevation myocardial infarction (STEMI), and severe hyperglycemia significantly contributes to increased mortality and morbidity in this population [9]. Conversely, hypoglycemia has also been associated with elevated odds of mortality, arrhythmias, and other comorbidities in hospitalized STEMI patients, regardless of DM status [10]. Additionally, recent studies have highlighted glycemic variability (GV) as a crucial aspect of dysglycemia [11], garnering considerable attention in the academic literature. GV not only serves as a reflection of glycemic control throughout the progression of the disease, but also serves as a predictive indicator for the occurrence of hyperglycemia or hypoglycemia [12]. An increasing number of studies have indicated that GV plays a pivotal role in determining adverse outcomes, with findings suggesting that it is a strong predictor of mortality and in-hospital complications among patients with STEMI, regardless of their DM status [13–15]. However, these studies included different populations and did not reflect the overall effect of acute GV on STEMI. Therefore, this study aimed to explore the impact of acute GV on short-term outcomes in Chinese patients with STEMI.

## Methods

### Study design and patient selection

This observational multicenter study, which was conducted at 274 centers in China between June 2001 and July 2004, enrolled 7510 consecutive patients diagnosed with acute STEMI who were admitted within 12 h of symptom onset. The diagnostic criteria for acute STEMI included typical chest pain or ischemic symptoms; dynamic changes in the electrocardiogram (ECG) showing elevation of ST-segment more than 0.2 mV in two adjacent leads (V1, V2, and V3) or more than 0.1 mV in other leads; new left bundle branch block (LBBB); and elevated levels of cardiac injury biomarkers (troponin I and creatine kinase MB). The exclusion criteria consisted of advanced malignancies or other diseases limiting life expectancy less than 1 month, and an inability to finish the follow-up period. The ethics committees at Fuwai Hospital and each participating center approved the study protocols in accordance with the Declaration of Helsinki. All patients provided written consent.

After hospitalization, patients received treatment according to clinical guidelines applicable at the time of the study and local healthcare standards for managing STEMI. Reperfusion treatment, such as primary percutaneous coronary intervention (PCI) or thrombolytic therapy, was utilized. Primary PCI procedures were conducted through radial or femoral artery access using established techniques by experienced cardiologists at specialized facilities. A total of 374 patients were excluded due to incomplete data, leaving 7136 patients for inclusion in the final analysis of the study.

### Data collection and laboratory measurements

Upon admission, main demographic, clinical, and laboratory characteristics, as well as key data about drugs, were collected. The admission Thrombolysis in Myocardial Infarction Risk Score (TRS) was determined utilizing a scoring system that assigned points based on specific criteria [16]. Furthermore, detailed laboratory information was collected, such as three venous BG values within 24 h and glycosylated hemoglobin (HbA1c) levels at the time of admission. Experienced researchers thoroughly reviewed the medical records of patients and addressed any inconsistencies in data collection through consultation.

Venous blood samples were consistently obtained from the antecubital vein of participants using a 21-gauge sterile syringe and collected in either EDTA-treated or plain tubes for laboratory testing. Venous BG levels were assessed upon admission, as well as at 6 ± 2 h and 24 ± 2 h post-admission, utilizing the glucose oxidase method for each patient. Blood samples were obtained from the cubital vein upon admission for the HbA1c test and then sent to the central laboratory of Fuwai Hospital in Beijing, where high-performance liquid chromatography was used. Consistent with standardized protocols, all participating centers adhered to identical blood collection procedures and testing methodologies. DM was characterized by an HbA1c level of ≥ 6.5%, whereas non-DM was indicated by an HbA1c level of < 6.5%.

Admission blood glucose (ABG) was assessed according to the BG levels of patients within 6 ± 2 h of admission. The mean blood glucose (MBG) level was computed as the average of three BG values recorded within 24 h after admission for each patient. The standard deviation (SD) of MBG was calculated as follows:

SD = √[((ABG – MBG)^2^ + (BG at 6 ± 2 h – MBG)^2^ + (BG at 24 ± 2 h – MBG)^2^)/3]. GV was assessed using the coefficient of variation of the BG level, calculated as MBG divided by SD for each patient, reflecting the degree of BG fluctuation within the 24-hour period.

### Study outcomes and follow-up

All patients underwent a 30-day follow-up period, which involved interviews conducted in the clinic, telephone conversations with patients or their family members, or a review of medical documentation. The primary outcome was all-cause death within 30 days of enrollment, and the secondary outcome was major adverse cardiovascular events (MACEs). MACEs were evaluated as a composite outcome, encompassing all-cause death, cardiac arrest, cardiogenic shock, reinfarction, stroke, and major bleeding. Trained research personnel, unaware of the study’s objectives, carried out the assessment of these outcome events.

All-cause death included deaths from cardiovascular and non-cardiovascular causes. Cardiac arrest was defined as ventricular fibrillation or pulseless ventricular tachycardia, or scenarios of clinical pulselessness characterized by pulseless electrical activity or bradycardia necessitating cardiopulmonary resuscitation and/or emergency defibrillation. Cardiogenic shock was identified by the presence of systolic arterial hypotension (< 90 mmHg) persisting for more than 30 min, along with symptoms of inadequate blood flow that did not improve with fluid adjustments, necessitating the use of intravenous drugs or mechanical devices to support BP. Reinfarction was characterized by recurrent typical chest pain accompanied by new ischemic ECG changes (such as ST-segment re-elevation, depression, or new Q waves), and further elevation in enzyme levels (reaching double the normal upper limit if they had previously returned to normal, or increasing by 50% if already elevated).

### Statistical analysis

Continuous variables are expressed as the means ± SD for data that followed a normal distribution, or as quartiles for data that did not follow a normal distribution, with normality assessed using the Kolmogorov-Smirnov test. Categorical variables are presented as frequencies (percentages). Group comparisons utilized analysis of variance or the Mann-Whitney U test for continuous variables and Pearson’s chi-square test or Fisher’s exact test for categorical variables. Outcome estimates were calculated using the Kaplan-Meier method and analyzed with the log-rank test. Cox proportional hazards models were used for outcome analysis. Well-established risk factors and variables with *p* < 0.05 were included in multivariable analyses. Adjusted hazard ratios (HRs) and their respective 95% confidence intervals (CIs) were calculated relative to the reference group, where HRs was set to 1. Statistical significance was set at *p* < 0.05, and all tests were two-sided. Statistical analysis was conducted with SPSS 26.0 (IBM Corporation, New York, NY, USA) and GraphPad Prism 9.0 (GraphPad Software, Boston, MA, USA).

## Results

### Baseline characteristics

A total of 7136 patients with primary STEMI diagnosis and with complete data were included in the present study. The average age of all patients was 62.5 ± 11.9 years old, average weight of all patients was 66.7 ± 11.8 kg, and the mean systolic BP(SBP) and diastolic BP (DBP) were 126.2 ± 26.1 and 78.8 ± 16.6 mmHg, respectively. The mean heart rate was (77.2 ± 18.5) beat/minute, the average ABG was (8.5 ± 4.2) mmol/L, the average HbA1c was 6.0 ± 1.1%, and the average GV was 22 ± 16% (Table 1).

**Table 1:** Baseline characteristics and treatment of STEMI patients according to GV tertiles

	All patients *N* = 7136	GV1(≤ 12.8%) *N* = 2453	GV2(12.8%~26.3%) *N* = 2361	GV3(≥ 26.3%) *N* = 2322	*P* value
Age(years)	62.5 ± 11.9	62.0 ± 12.3	62.4 ± 11.8	63.3 ± 11.5	0.001
Female, n(%)	2041 (28.6)	668 (27.2)	646 (27.4)	727 (31.3)	0.002
Weight(kg)	66.7 ± 11.8	66.5 ± 11.6	67.2 ± 11.8	66.3 ± 11.9	0.038
SBP(mmHg)	126.2 ± 26.1	126.7 ± 24.6	126.8 ± 25.6	125.2 ± 28.0	0.063
DBP(mmHg)	78.8 ± 16.6	79.0 ± 15.4	79.1 ± 16.4	78.1 ± 17.8	0.060
HR(bpm)	77.2 ± 18.5	77.5 ± 17.5	76.9 ± 18.3	77.2 ± 19.8	0.568
Anterior STE or LBBB	3771 (52.8)	1350 (55.0)	1249 (52.9)	1172 (50.5)	0.007
Time to treatment >4 h	4686 (65.7)	1643 (67.0)	1556 (65.9)	1487 (64.0)	0.097
TRS	4.2 ± 2.4	4.1 ± 2.4	4.1 ± 2.3	4.4 ± 2.4	< 0.001
**Histories**					
Previous myocardial infarction	567 (7.9)	178 (7.3)	192 (8.1)	197 (8.5)	0.269
Previous heart failure	190 (2.7)	67 (2.7)	53 (2.2)	70 (3.0)	0.253
Hypertension	2886 (40.4)	939 (38.3)	966 (40.9)	981 (42.2)	0.017
DM	1683 (23.6)	503 (20.5)	579 (24.5)	601 (25.9)	< 0.001
Previous stroke	668 (9.4)	179 (7.3)	219 (9.3)	270 (11.6)	< 0.001
Laboratory tests					
Admission blood glucose(mmol/L)	8.5 ± 4.2	7.3 ± 2.8	8.4 ± 3.5	10.0 ± 5.5	< 0.001
HbA1c(%)	6.0 ± 1.1	6.0 ± 1.0	6.0 ± 1.1	6.1 ± 1.1	0.062
GV(%)	22 ± 16	7 ± 3	19 ± 4	41 ± 14	< 0.001
Hemoglobin(g/L)	135.8 ± 20.3	135.3 ± 18.9	136.7 ± 20.2	135.5 ± 21.7	0.030
**Reperfusion therapy**					
Thrombolytic therapy	3752 (52.6)	1185 (48.3)	1265 (53.6)	1302 (56.1)	< 0.001
Primary PCI	850 (11.9)	281 (11.5)	277 (11.7)	292 (12.6)	0.464
**Medications**					
Antiplatelet therapy	6923 (97.0)	2363 (96.3)	2301 (97.5)	2259 (97.3)	0.046
Statins	5179 (72.6)	1791 (73.0)	1702 (72.1)	1686 (72.6)	0.785
β-blockers	4466 (62.6)	1507 (61.4)	1502 (63.6)	1457 (62.7)	0.281
ACEIs/ARBs	5204 (72.9)	1744 (71.1)	1745 (73.9)	1715 (73.9)	0.041
Nitrate	6587 (92.3)	2278 (92.9)	2193 (92.9)	2116 (91.1)	0.032
CCBs	930 (13.0)	380 (15.5)	281 (11.9)	269 (11.6)	< 0.001
Diuretics	1835 (25.7)	594 (24.2)	586 (24.8)	655 (28.2)	0.003
Insulin	1075 (15.1)	290 (11.8)	355 (15.0)	430 (18.5)	< 0.001

STEMI, ST-segment elevation myocardial infarction; GV, glycemic variability; SBP, systolic blood pressure; DBP, diastolic blood pressure; HR, heart rate; TRS, TIMI risk score; STE, ST-segment elevation; LBBB, left bundle branch block; DM, diabetes mellitus; PCI, percutaneous coronary intervention; ACEIs, angiotensin-converting enzyme inhibitors; ARBs, angiotensin receptors blockers; CCBs, calcium channel blocker

Patients were stratified into three groups based on tertiles of GV: GV1(≤ 12.8%), GV2 ($12.8\% \sim 26.3\%$), and GV3(≥ 26.3%). Older age, female sex, higher TRS, history of hypertension, DM, and previous stroke were most frequent in the highest tertile (GV3 group). ABG values were also higher in the GV3 group, while HbA1c did not show significantly different.

### Clinical outcomes based on GV tertiles and DM status

**Fig. 1:**
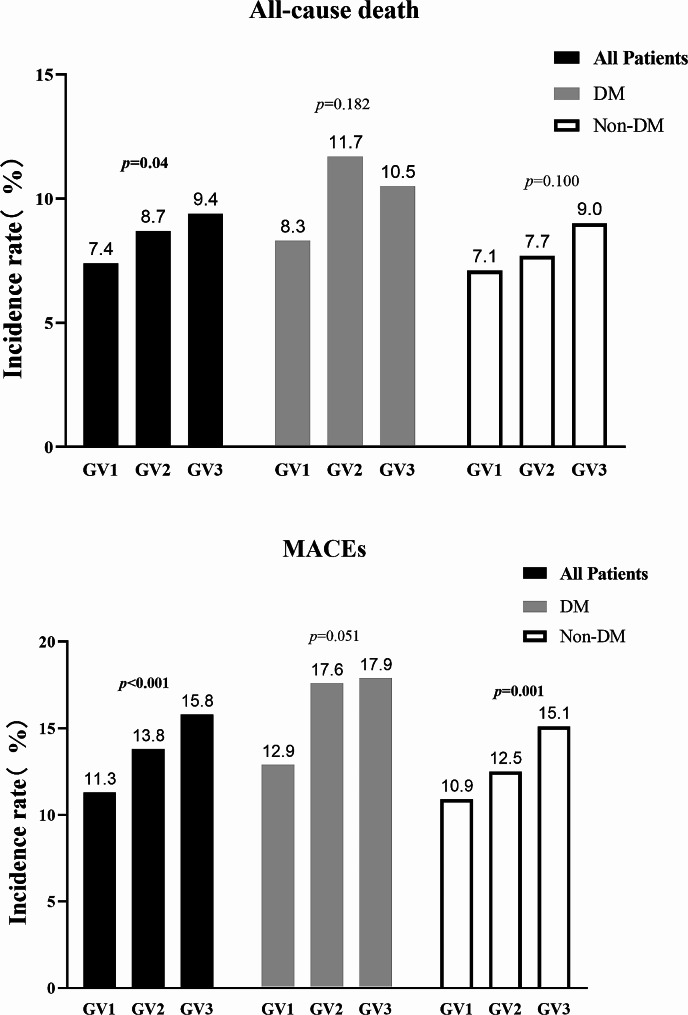
Event rates of outcome variables in patients with STEMI by GV categories and with or without DM status

During the 30-day follow-up period, a total of 604(8.5%) all-cause deaths and 970(13.6%) MACEs were recorded. The incidence of both all-cause death and MACEs showed a significant increase with higher GV tertiles, with 30-days mortality rates of GV1 was 7.4%, and 8.7% for GV2 and 9.4% for GV3 (*p* = 0.004), and MACEs incidence rate was 11.3%, 13.8% and 15.8% for the GV1, GV2 and GV3 groups, respectively (*p* < 0.001) (Table 2). This trend was observed in the DM and non-DM groups, with patients in the DM group showing a worse prognosis compared to those in the non-DM group, although the difference was not statistically significant (Fig. 1).

**Table 2:** Clinical outcomes of STEMI patients according to GV tertiles

	All patients *N* = 7136	GV1(≤ 12.8%) *N* = 2453	GV2(12.8%~26.3%) *N* = 2361	GV3(≥ 26.3%) *N* = 2322	*P* value
All-cause death	604 (8.5)	181 (7.4)	205 (8.7)	218 (9.4)	0.040
Reinfarction	143 (2.0)	39 (1.6)	50 (2.1)	54 (2.3)	0.172
Cardiac shock	394 (5.5)	109 (4.4)	136 (5.8)	149 (6.4)	0.010
Cardiac arrest	340 (4.8)	101 (4.1)	116 (4.9)	123 (5.3)	0.147
Stroke	62 (0.9)	18 (0.7)	20 (0.8)	24 (1.0)	0.531
Major bleeding	23 (0.3)	4 (0.2)	11 (0.5)	8 (0.3)	0.175
MACEs	970 (13.6)	278 (11.3)	325 (13.8)	367 (15.8)	< 0.001

STEMI, ST-segment elevation myocardial infarction; GV, glycemic variability; MACEs, major adverse cardiovascular events

**Fig. 2:**
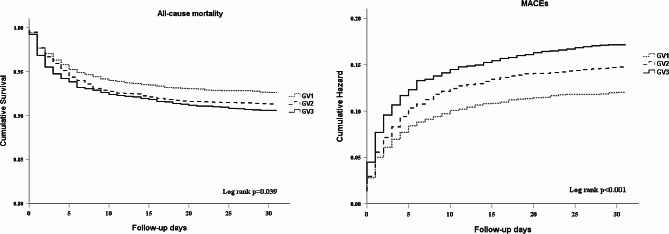
The Kaplan-Meyer survival curves and event rates for all-cause death and MACEs in patients with STEMI according to the GV categories

The Kaplan-Meier curves depicting 30-day mortality and MACEs showed significant differences among groups (*p* < 0.001), with a notably elevated cumulative mortality rate observed in GV3 compared to the other groups (Fig. 2). Similarly, the cumulative occurrence of MACEs within 30-day was significantly higher in the GV3 group compared to the other groups (Fig. 2).

### Association of GV with 30-day outcomes using multivariate Cox analysis

In Table 3, the multivariate Cox regression models show the factors associated with 30-day mortality and MACEs. In these models, GV was considered as a continuous variable, and with variables with *p* < 0.05 in univariate analysis and HbA1c all being taken into account for adjustments (supplement Table S1). GV was independently associated with an increased risk of all-cause mortality (HR 1.679, 95% CI 1.005–2.804) and MACEs (HR 2.064, 95% CI 1.386–3.074) (Table 3). Furthermore, older age, female sex, lower SBP, higher heart rate, hypertension, anterior STE of the LBBB, and stroke were linked to increased risks of both all-cause mortality and MACEs. Conversely, treatment with primary PCI, angiotensin-converting enzyme inhibitors (ACEIs)/angiotensin receptor antagonists (ARBs), β-blockers, and statins were associated with a reduced risk of all-cause mortality and MACEs.

**Table 3:** Predictors of all-cause mortality and MACEs by multivariate Cox analysis

	All-cause death		MACEs	
	HR (95% CI)	P value	HR (95% CI)	P value
GV	1.679 (1.005–2.804)	0.048	2.064(1.386–3.074)	< 0.001
Age	1.055 (1.044–1.066)	< 0.001	1.035(1.027–1.043)	< 0.001
Female	1.505 (1.214–1.865)	< 0.001	1.253(1.055–1.489)	< 0.001
Weight, Kg	1.001 (0.992–1.010)	0.865	0.997(0.990–1.004)	0.450
SBP, mmHg	0.983 (0.976–0.989)	< 0.001	0.985(0.980–0.990)	< 0.001
DBP, mmHg	1.010 (1.000-1.020)	0.059	1.001(0.993–1.008)	0.879
Heart rate, bpm	1.014 (1.010–1.018)	< 0.001	1.011(1.008–1.015)	< 0.001
Anterior STE or LBBB	1.602 (1.298–1.976)	< 0.001	1.197(1.019–1.407)	0.028
Time to treatment > 4 h	1.333(1.070–1.660)	0.010	1.016(0.865–1.193)	0.848
Previous myocardial infarction	1.184 (0.868–1.614)	0.286	1.040(0.803–1.347)	0.766
Previous heart failure	1.229 (0.833–1.815)	0.299	1.231(0.875–1.732)	0.232
DM	1.056 (0.781–1.429)	0.722	1.170(0.922–1.485)	0.196
Hypertension	1.357 (1.101–1.673)	0.004	1.242(1.055–1.463)	0.009
Stroke	1.363 (1.047–1.774)	0.021	1.406(1.138–1.739)	0.002
HbA1c	0.958 (0.844–1.089)	0.514	0.954(0.863–1.054)	0.354
Thrombolytic therapy	1.084 (0.884–1.330)	0.437	1.199(1.019–1.410)	0.028
Primary PCI	0.366 (0.219–0.612)	< 0.001	0.686(0.505–0.930)	0.015
Antiplatelet therapy	0.762 (0.533–1.089)	0.135	0.816(0.599–1.112)	0.198
β-blockers	0.621 (0.507–0.762)	< 0.001	0.642(0.546–0.754)	< 0.001
ACEIs/ARBs	0.641 (0.516–0.795)	< 0.001	0.742(0.627–0.878)	< 0.001
Statins	0.585 (0.477–0.716)	< 0.001	0.650(0.553–0.763)	< 0.001

STEMI, ST-segment elevation myocardial infarction; GV, glycemic variability; MACEs, major adverse cardiovascular events; HR, hazards ratio; CI, confidence interval; SBP, systolic blood pressure; DBP, diastolic blood pressure; STE, ST-segment elevation; LBBB, left bundle branch block; DM, diabetes mellitus; PCI, percutaneous coronary intervention; ACEIs, angiotensin-converting enzyme inhibitors; ARBs, angiotensin receptors blockers

**Fig. 3:**
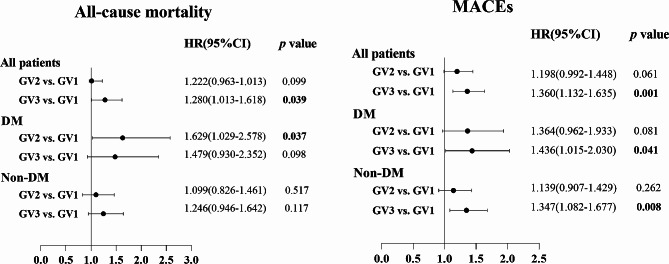
Multivariate Cox proportional models of predictors for all-cause death and MACEs by GV categories and with or without DM status

Figure 3 illustrates the associations between GV tertiles and all-cause mortality as well as MACEs based on DM status. After adjusting for confounding factors in the multivariate Cox regression models, the GV2 group exhibited an independent association with all-cause mortality in the DM subgroup, while no significant difference in GV was observed in the non-DM subgroup. Regarding MACEs, irrespective of whether considering all patients, the DM subgroup, or the non-DM subgroup, the GV3 group demonstrated a significant increase in risk for MACEs.

## Discussion

In this study, we aimed to explore the impact of acute GV on short-term outcomes in Chinese patients with acute STEMI. The main findings are outlined as follows. Firstly, patients with varying levels of GV demonstrated distinctive clinical features. Patients with higher GV levels tended to be older, exhibit with more severe clinical characteristics, and have a higher prevalence of comorbidities along with increased BG fluctuations compared to those with lower GV levels. Secondly, we observed that a higher GV level during hospitalization was associated with a significantly elevated risk of short-term all-cause mortality and MACEs in Chinese STEMI patients. Importantly, this association was consistent across both the DM and non-DM groups. Lastly, our multivariate Cox regression analysis showed that GV was an independent predictor of higher risk for MACEs, and importantly, this association was not influenced by the presence of DM. Although the enrollment period of our study reflects a time gap, our study’s focus on GV and its implications for short-term outcomes in Chinese patients with STEMI remains relevant. These findings emphasize the significance of acute GV as a predictive indicator in patients with acute STEMI, highlighting its potential utility in risk stratification and clinical management irrespective of DM status.

More and more recent studies have focused on GV and its association with the prognosis of coronary artery disease, and a meta-analysis indicated that increased GV may be associated with poor prognosis in coronary artery disease patients regardless of DM status [17]. Similarly, another meta-analysis suggested that increased GV is correlated with poorer prognosis in acute coronary syndrome patients [18]. Specifically for acute myocardial infarction, Zhang et al. first demonstrated that GV serves as an independent predictor for composite 30-day MACEs in DM patients with STEMI undergoing primary PCI [14]. Yi et al. also found that higher GV is associated with an increased risk of 30-day MACE in DM patients with STEMI receiving PCI [19]. Moreover, for non-DM patients with STEMI treated with PCI, GV was identified as a predictor of short-term MACEs and mortality [13]. According to the results of these studies, GV may have a greater impact on prognosis than previously thought, supporting the ongoing relevance of GV assessment in contemporary clinical practice. Consistent with these findings, our study also revealed that acute elevation in GV levels was significantly associated with higher risks of 30-day all-cause mortality and MACEs in Chinese STEMI patients. Importantly, GV emerged as an independent predictor of increased MACEs risk, a relationship unaffected by DM status and reperfusion therapy.

Increased GV has been associated with different cardiovascular diseases and adverse outcomes across different patient populations. In the DM population, higher GV has been associated with an increased risk of decrease in estimated glomerular filtration rate [20]. In DM and acute HF patients, higher GV was identified as the strongest independent predictor for mid-term MACEs [21]. In patients undergoing cardiac surgery, high acute GV has been linked to poor in-hospital outcomes [22, 23]. Moreover, in the adult population, high GV associated with increased risk of atrial fibrillation [24]. The acute GV, reflecting the physiological stress response during the initial hospitalization period, has been proposed as a predictor of prognosis in patients with acute diseases. Elevated acute GV has been recognized as a potential predictor of poor survival in patients with sepsis [25]. High GV has also been associated with adverse outcomes in critically ill patients [26, 27], including being an independent risk factor for in-hospital mortality in intensive care unit patients, partly due to an increased risk of ventricular arrhythmias [28]. Elevated acute GV could be an indicator of unfavorable functional results and death in patients with intracerebral hemorrhage [29].These results underscore the significance of monitoring and managing GV in various clinical settings to improve patient outcomes and reduce the risk of adverse outcomes.

The potential mechanisms underlying the adverse effects of acute GV on poor prognosis in patients with STEMI remain incompletely understood. Studies on mechanistic pathways have indicated that high GV leads to various adverse outcomes, such as increased inflammation, oxidative stress, and apoptosis, all of which contribute to endothelial dysfunction, a critical factor in the pathogenesis of cardiovascular complications [4, 30–32]. GV has been shown to have negative effects on autonomic function and increase the thrombotic properties of platelets, which can contribute to the development of macrovascular disease [33]. In a hyperglycemic environment, the thrombotic properties of platelets are further heightened, potentially leading to additional cardiovascular complications [31]. Additionally, hypoglycemia may serve as another potential association between GV and worse cardiovascular endpoints. Studies have indicated that higher GV levels are associated with more frequent hypoglycemic episodes, which in turn can predict all-cause mortality in patients with DM. Hypoglycemia has the potential to trigger the occurrence of MACEs by eliciting inflammatory responses, abnormal blood coagulation, sympathetic-adrenal responses, and endothelial dysfunction [34].

The practical significance of assessing GV as a predictor of outcomes in STEMI patients lies in its potential to identify individuals at higher risk of adverse events who may benefit from targeted interventions to optimize glycemic control. Moreover, emerging research has highlighted the potential of personalized glycemic management strategies targeting GV reduction to improve outcomes in high-risk patient populations, underscoring the translational implications of our study’s findings. Therefore, reducing short-term GV may emerge as a priority during the acute phase. Research conducted by Hanajima et al. indicated that GV plays a significant role in alterations to left ventricular structure and function post-STEMI, with lower GV potentially promoting left ventricular reverse remodeling and improving prognosis [35]. Previous studies have shown that physical activities can reduce GV in DM patients [36, 37], while Vijayakumar et al. demonstrated that regular short-term yoga practice could significantly reduce GV in patients with DM [38]. Furthermore, a review has suggested that administering a preoperative carbohydrate load may effectively mitigate metabolic disturbances such as GV, thereby potentially reducing postoperative morbidity and mortality [39]. Additionally, sodium-glucose cotransporter 2 inhibitors represent a promising therapeutic approach for reducing GV [40]. This collective evidence underscores the significance of effective clinical management in improving outcomes for STEMI patients.

## Limitations

This study had some limitations. Firstly, we use coefficient of variation of blood glucose for GV in our study, this may not the best index. Although GV is important clinically, there remains no consensus regarding its definition or the most appropriate index for its assessment. Secondly, the measurement of GV in our study relied on all available blood glucose test results rather than continuous glucose monitoring, potentially limiting accuracy compared to GV assessment with continuous glucose monitoring, therefore, it should be noted that the study may have used somewhat inaccurate GV. Thirdly, the enrollment period of our study spanning from June 2001 to July 2004 indeed reflects a significant time gap since the study was conducted, however, despite the temporal gap, this study’s focus on GV and its implications for short-term outcomes in Chinese patients with STEMI remains relevant. Fourthly, as our study retrospectively analyzed the importance of GV for short-term outcomes in STEMI patients, and it is possible that the presence of confounders and selection bias could have impacted the results. Lastly, our focus on short-term prognosis over a 30-day period in acute STEMI patients necessitates further investigation into the impact of GV on long-term outcomes to validate our findings.

## Conclusion

Our study findings indicate that a high GV level during hospitalization was significantly associated with an increased risk of 30-day all-cause mortality and MACEs in Chinese patients with STEMI. Moreover, GV emerged as an independent predictor of increased MACEs risk, and was unaffected by the presence of DM. These findings underscore the significance of glycemic control strategies in improving cardiovascular outcomes, highlighting the potential for GV monitoring to be used in the risk stratification and clinical management of patients with STEMI.

### Supplementary Information

**Additional file 1: Table S1. **Predictors of all-cause mortality and MACEs by univariate Cox analysis.

## Data Availability

No datasets were generated or analysed during the current study.

## Abbreviations

GV: Glycemic variability
BG: Blood glucose
ABG: Admission blood glucose
MBG: Mean blood glucose
HbA1c: Glycosylated hemoglobin
STEMI: ST-segment elevation myocardial infarction
MI: Myocardial infarction
PCI: Percutaneous coronary intervention
HF: Heart failure
DM: Diabetes mellitus
MACEs: Major adverse cardiac events
TRS: TIMI risk score
BP: Blood pressure
ECG: Electrocardiogram
ACEIs: Angiotensin-converting enzyme inhibitors
ARBs: Angiotensin receptor antagonists
CCBs: Calcium channel blockers
HRs: Hazard ratios
CIs: Confidence intervals
SD: Standard deviation

## References

[1] Ceriello A, Monnier L, Owens D (2019). Glycaemic variability in diabetes: clinical and therapeutic implications. LANCET DIABETES ENDO.

[2] Rodbard D (2018). Glucose variability: a review of clinical applications and Research Developments. DIABETES TECHNOL THE.

[3] Wu N, Shen H, Liu H, Wang Y, Bai Y, Han P (2016). Acute blood glucose fluctuation enhances rat aorta endothelial cell apoptosis, oxidative stress and pro-inflammatory cytokine expression in vivo. CARDIOVASC DIABETOL.

[4] Valente T, Arbex AK (2021). Glycemic variability, oxidative stress, and impact on complications related to type 2 diabetes Mellitus. CURR DIABETES REV.

[5] Hsu JC, Yang YY, Chuang SL, Huang KC, Lee JK, Lin LY. Long-term visit‐to‐visit glycemic variability as a predictor of major adverse Limb and Cardiovascular events in patients with diabetes. J AM HEART ASSOC 2023, 12(3). 10.1161/JAHA.122.025438 PMC9973660 36695326

[6] Ren X, Wang Z, Guo C. Long-term glycemic variability and risk of stroke in patients with diabetes: a meta-analysis. DIABETOL METAB SYNDR 2022, 14(1). 10.1186/s13098-021-00770-0 PMC8756678 35022087

[7] Kapłan C, Kalemba A, Krok M, Krzych A (2022). Effect of Treatment and Nutrition on Glycemic Variability in critically ill patients. Int J Environ Res Public Health.

[8] Belli M, Bellia A, Sergi D, Barone L, Lauro D, Barillà F (2023). Glucose variability: a new risk factor for cardiovascular disease. ACTA DIABETOL.

[9] Stalikas N, Papazoglou AS, Karagiannidis E, Panteris E, Moysidis D, Daios S, Anastasiou V, Patsiou V, Koletsa T, Sofidis G (2022). Association of stress induced hyperglycemia with angiographic findings and clinical outcomes in patients with ST-elevation myocardial infarction. CARDIOVASC DIABETOL.

[10] Humos B, Mahfoud Z, Dargham S, Al SJ, Jneid H, Abi KC (2022). Hypoglycemia is associated with a higher risk of mortality and arrhythmias in ST-elevation myocardial infarction, irrespective of diabetes. FRONT CARDIOVASC MED.

[11] Monnier L, Colette C (2008). Glycemic variability: should we and can we prevent it?. Diabetes Care.

[12] Zinman B, Marso SP, Poulter NR, Emerson SS, Pieber TR, Pratley RE, Lange M, Brown-Frandsen K, Moses A, Ocampo FA (2018). Day-to-day fasting glycaemic variability in DEVOTE: associations with severe hypoglycaemia and cardiovascular outcomes (DEVOTE 2). Diabetologia.

[13] Mi S, Su G, Yang H, Zhou Y, Tian L, Zhang T, Tao H. Comparison of in-hospital glycemic variability and admission blood glucose in predicting short-term outcomes in non-diabetes patients with ST elevation myocardial infarction underwent percutaneous coronary intervention. DIABETOL METAB SYNDR 2017, 9(1). 10.1186/s13098-017-0217-1 PMC5359987 28344659

[14] Zhang JW, He LJ, Cao SJ, Yang Q, Yang SW, Zhou YJ (2014). Effect of glycemic variability on short term prognosis in acute myocardial infarction subjects undergoing primary percutaneous coronary interventions. DIABETOL METAB SYNDR.

[15] Yang CD, Shen Y, Ding FH, Yang ZK, Hu J, Shen WF, Zhang RY, Lu L, Wang XQ (2020). Visit-to-visit fasting plasma glucose variability is associated with left ventricular adverse remodeling in diabetic patients with STEMI. CARDIOVASC DIABETOL.

[16] Morrow DA, Antman EM, Charlesworth A, Cairns R, Murphy SA, de Lemos JA, Giugliano RP, McCabe CH, Braunwald E (2000). TIMI risk score for ST-elevation myocardial infarction: a convenient, bedside, clinical score for risk assessment at presentation: an intravenous nPA for treatment of infarcting myocardium early II trial substudy. Circulation.

[17] Pu Z, Lai L, Yang X, Wang Y, Dong P, Wang D, Xie Y, Han Z (2020). Acute glycemic variability on admission predicts the prognosis in hospitalized patients with coronary artery disease: a meta-analysis. Endocrine.

[18] Zhang L, Li F, Liu H, Zhang Z, Yang F, Qian L, Wang R (2022). Glycaemic variability and risk of adverse cardiovascular events in acute coronary syndrome. Diabetes Vascular Disease Res.

[19] Yi M, Cao Q, Tang WH, Liu Q, Ke X (2022). Day-to‐day fasting plasma glucose variability on the short‐term prognosis of ST‐segment elevation myocardial infarction: a retrospective cohort study. CLIN CARDIOL.

[20] Deravi N, Sharifi Y, Koohi F, Zadeh SST, Masrouri S, Azizi F, Hadaegh F. The association between fasting plasma glucose variability and incident eGFR decline: evidence from two cohort studies. BMC Public Health 2023, 23(1). 10.1186/s12889-023-15463-8 PMC10041700 36973769

[21] Gerbaud E, De La Bouchard A, Baudinet T, Montaudon M, Beauvieux M, Lemaître A, Cetran L, Seguy B, Picard F, Vélayoudom F (2022). Glycaemic variability and hyperglycaemia as prognostic markers of Major Cardiovascular events in Diabetic patients Hospitalised in Cardiology Intensive Care Unit for Acute Heart failure. J CLIN MED.

[22] Chang S, Xu M, Wang Y, Zhang Y. Acute Glycemic variability and early outcomes after cardiac surgery: a Meta-analysis. HORM METAB RES; 2023. 10.1055/a-2106-5539 37402380

[23] Zheng Q, Liu X, Lan H, Guo Q, Xiong T, Wang K, Jiang C, Zhang J, Wang G, Dong N (2023). Association of fasting blood glucose variability with all-cause mortality in heart transplant recipients. CLIN Transpl.

[24] Li W, Wang Y, Zhong G. Glycemic variability and the risk of atrial fibrillation: a meta-analysis. FRONT ENDOCRINOL 2023, 14. 10.3389/fendo.2023.1126581 PMC10232736 37274320

[25] Li X, Zhang D, Chen Y, Ye W, Wu S, Lou L, Zhu Y. Acute glycemic variability and risk of mortality in patients with sepsis: a meta-analysis. DIABETOL METAB SYNDR 2022, 14(1). 10.1186/s13098-022-00819-8 PMC9034073 35461267

[26] Kim SH, Kim JY, Kim ES, Park IR, Ha EY, Chung SM, Moon JS, Yoon JS, Won KC, Lee HW (2022). Early glycaemic variability increases 28-day mortality and prolongs intensive care unit stay in critically ill patients with pneumonia. ANN MED.

[27] Dong M, Liu W, Luo Y, Li J, Huang B, Zou Y, Liu F, Zhang G, Chen J, Jiang J et al. Glycemic variability is independently Associated with Poor Prognosis in five Pediatric ICU centers in Southwest China. FRONT NUTR 2022, 9. 10.3389/fnut.2022.757982 PMC8905539 35284444

[28] Su Y, Fan W, Liu Y, Hong K. Glycemic variability and in-hospital death of critically ill patients and the role of ventricular arrhythmias. CARDIOVASC DIABETOL 2023, 22(1). 10.1186/s12933-023-01861-0 PMC10258982 37308889

[29] Jiao X, Wang H, Li M, Lu Y (2023). Glycemic variability and prognosis of patients with Intracerebral Hemorrhage: a Meta-analysis. HORM METAB RES.

[30] Lu J, Wang C, Cai J, Shen Y, Chen L, Zhang L, Lu W, Zhu W, Hu G, Xia T (2021). Association of HbA1c with all-cause Mortality Across varying degrees of glycemic variability in type 2 diabetes. J CLIN ENDOCR METAB.

[31] Alfieri V, Myasoedova VA, Vinci MC, Rondinelli M, Songia P, Massaiu I, Cosentino N, Moschetta D, Valerio V, Ciccarelli M (2021). The role of Glycemic Variability in Cardiovascular disorders. Int J Mol Sci.

[32] Yamazaki M, Hasegawa G, Majima S, Mitsuhashi K, Fukuda T, Iwase H, Kadono M, Asano M, Senmaru T, Tanaka M (2014). Effect of repaglinide versus glimepiride on daily blood glucose variability and changes in blood inflammatory and oxidative stress markers. DIABETOL METAB SYNDR.

[33] Sun B, Luo Z, Zhou J (2021). Comprehensive elaboration of glycemic variability in diabetic macrovascular and microvascular complications. CARDIOVASC DIABETOL.

[34] Amiel SA, Aschner P, Childs B, Cryer PE, de Galan BE, Frier BM, Gonder-Frederick L, Heller SR, Jones T, Khunti K (2019). Hypoglycaemia, cardiovascular disease, and mortality in diabetes: epidemiology, pathogenesis, and management. Lancet Diabetes Endocrinol.

[35] Hanajima Y, Iwahashi N, Kirigaya J, Horii M, Minamimoto Y, Gohbara M, Abe T, Okada K, Matsuzawa Y, Kosuge M et al. Prognostic importance of glycemic variability on left ventricular reverse remodeling after the first episode of ST-segment elevation myocardial infarction. CARDIOVASC DIABETOL 2023, 22(1). 10.1186/s12933-023-01931-3 PMC10403862 37542320

[36] Bennetsen SL, Feineis CS, Legaard GE, Lyngbæk M, Karstoft K, Ried-Larsen M (2020). The impact of physical activity on glycemic variability assessed by continuous glucose monitoring in patients with type 2 diabetes Mellitus: a systematic review. FRONT ENDOCRINOL.

[37] Jaggers JR, King KM, McKay T, Dyess RJ, Thrasher BJ, Wintergerst KA (2023). Association between intensity levels of physical activity and glucose variability among children and adolescents with type 1 diabetes. Int J Environ Res Public Health.

[38] Vijayakumar V, Mavathur R, Kannan S, Sharma MNK, Raguram N, Kuppusamy M (2023). Effect of yoga on reducing glycaemic variability in individuals with type 2 diabetes: a randomised controlled trial. DIABETES METAB.

[39] Canelli R, Louca J, Hartman C, Bilotta F (2023). Preoperative carbohydrate load to reduce perioperative glycemic variability and improve surgical outcomes: a scoping review. World J Diabetes.

[40] Færch K, Blond MB, Bruhn L, Amadid H, Vistisen D, Clemmensen K, Vainø C, Pedersen C, Tvermosegaard M, Dejgaard TF (2021). The effects of dapagliflozin, metformin or exercise on glycaemic variability in overweight or obese individuals with prediabetes (the PRE-D trial): a multi-arm, randomised, controlled trial. Diabetologia.

