# Supplementary material for: Impact of acute glycemic variability on short-term outcomes in patients with ST-segment elevation myocardial infarction: a multicenter population-based study
Source: Cardiovasc Diabetol. 2024 May 7;23:155. doi: 10.1186/s12933-024-02250-x (PMC11077764; doi:10.1186/s12933-024-02250-x)
Supplement: Supplementary file 1 — Additional file 1: Table S1. Predictors of all-cause mortality and MACEs by univariate Cox analysis. [file 12933_2024_2250_MOESM1_ESM.docx]

**Supplement Table S1 Predictors of all-cause mortality and MACEs by univariate Cox analysis**

|  | **All-cause death** | | **MACEs** | |
| --- | --- | --- | --- | --- |
|  | HR(95%CI) | P value | HR(95%CI) | P value |
| GV | 2.021(1.292-3.163) | 0.002 | 2.564(1.815-3.621) | <0.001 |
| Age | 1.073(1.064-1.082) | <0.001 | 1.047(1.041-1.054) | <0.001 |
| Female | 2.212(1.885-2.596) | <0.001 | 1.735(1.026-1.972) | <0.001 |
| Weight, Kg | 0.971(0.964-0.978) | <0.001 | 0.978(0.972-0.983) | <0.001 |
| SBP, mmHg | 0.987(0.984-0.990) | <0.001 | 0.982(0.980-0.985) | <0.001 |
| DBP, mmHg | 0.982(0.977-0.986) | <0.001 | 0.975(0.971-0.979) | <0.001 |
| Heart rate, bpm | 1.019(1.015-1.023) | <0.001 | 1.011(1.007-1.014) | <0.001 |
| Anterior STE or LBBB | 1.379(1.172-1.624) | <0.001 | 1.003(1.884-1.138) | 0.961 |
| Time to treatment ＞4h | 1.483(1.237-1.777) | <0.001 | 1.057(1.010-1.326) | 0.035 |
| Previous myocardial infarction | 1.317(1.011-1.716) | 0.042 | 1.145(0.918-1.427) | 0.230 |
| Previous heart failure | 2.806(2.043-3.852) | <0.001 | 2.206(1.673-2.901) | <0.001 |
| DM | 1.315(1.102-1.568) | 0.002 | 1.295(1.126-1.489) | <0.001 |
| Hypertension | 1.238(1.055-1.453) | 0.009 | 1.008(0.958-1.235) | 0.196 |
| Stroke | 1.759(1.407-2.198) | <0.001 | 1.771(1.485-2.112) | <0.001 |
| HbA1c | 1.022(0.941-1.110) | 0.600 | 1.037(0.973-1.106) | 0.263 |
| Thrombolytic therapy | 0.725(0.618-1.851) | <0.001 | 0.908(0.800-1.029) | 0.131 |
| Primary PCI | 0.244(0.156-0.318) | <0.001 | 0.524(0.408-0.673) | <0.001 |
| Antiplatelet therapy | 0.354(0.261-0.479) | <0.001 | 0.431(0.332-0.559) | <0.001 |
| β-blockers | 0.477(0.406-0.559) | <0.001 | 0.499(0.440-0.566) | <0.001 |
| ACEIs/ARBs | 0.460(0.391-0.540) | <0.001 | 0.499(0.439-0.567) | <0.001 |
| Statins | 0.401(0.341-0.470) | <0.001 | 0.522(0.459-0.593) | <0.001 |

STEMI, ST-segment elevation myocardial infarction; GV, glycemic variability; MACEs, major adverse cardiovascular events; HR, hazards ratio; CI, confidence interval; SBP, systolic blood pressure; DBP, diastolic blood pressure; STE, ST-segment elevation; LBBB, left bundle branch block; DM, diabetes mellitus; PCI, percutaneous coronary intervention; ACEIs, angiotensin-converting enzyme inhibitors; ARBs, angiotensin receptors blockers.
